# Supplementary material for: Neurotoxicity of Chronic Co-Exposure of Lead and Ionic Liquid in Common Carp: Synergistic or Antagonistic?
Source: Int J Mol Sci. 2022 Jun 3;23(11):6282. doi: 10.3390/ijms23116282 (PMC9181186; doi:10.3390/ijms23116282)
Supplement: Supplementary file 1 [file ijms-23-06282-s001.zip › ijms-1722934-supplementary.pdf]

Neurotoxicity of chronic co-exposure of lead and ionic liquid on common carp: Synergistic or antagonism?

Weikai Ding [1,†], Yousef Sultan [1,2,†], Shumei Li [1], Wenjun Wen [1], Bangjun Zhang [1], Yiyi Feng [1], Junguo Ma [1] and Xiaoyu Li [1,*]

[1] Henan International Joint Laboratory of Aquatic Ecotoxicology and Health Protection, College of Life Science, Henan Normal University, Xinxiang, Henan, 453007, China

[2] Department of Food Toxicology and Contaminants, National Research Centre, Dokki, Cairo, 12622, Egypt

† These authors contributed equally to this work.

* Correspondence: 041035@htu.edu.cn

Supplementary Materials 1. *Pb and the ionic liquid contents in water*

In this study, the change in Pb and M8OI contents in treated water was measured within one day at 0, 4, 8, 12, 24 h following exposure. Pb was determined according to the method mentioned in the previous study by Rajeshkumar et al (Rajeshkumar et al., 2018).

The Concentration of the ionic liquid in water was determined using HPLC (Agilent 1200). The mobile phase was the mixture of acetonitrile and 25 mM of phosphate buffer (pH 3) with 0.5% triethylamine (20%, v/v). Column temperature was maintained at 30 °C. A volume of 10 μL was injected. The flow rate was controlled at 0.8 mL/min, and the detection wavelength was set at 212 nm. The M8OI content in water samples were estimated by HPLC with a C_{18} (150 × 4.6 mm, 5 mm) column（Agilent Technologies Inc.）.

Supplementary Materials 2. *Possible chemical reaction of Pb and the ionic liquid in the water*

A 10 mL Schlenk-tube was charged with M8OI (10 mg), Pb $(OAc)_2$ (10 mg) in H_2O (2 mL) with magnetic stirring under air atmosphere. The mixture was stirred for 24 h at room temperature. Then the reaction was extracted with DCM (3 × 10 mL), dried over anhydrous Na_2SO_4 and evaporated under the reduced pressure. The residue was detected by H-NMR (AVANCE III HD 600MHZ, Bruker, Switzerland).

Supplementary Materials 3. *M8OI content in common carp brain*

In this study, the brain from the treatment and control groups were collected to measure the contents of M8OI. The measuring method was carried out according to Habibul et al., (2021). In brief, brain samples were weighted, mixed and homogenized with 7 mL of 0.5% formic acid and methanol (5:2, v/v), and shaken overnight at room temperature to extract M8OI. After centrifugation at 10000 g for 30 min at room temperature, the supernatant extracts were collected to dry. And then, the powder contained M8OI was redissolved in 1 mL of methanol and filtrated with a 0.24 μm membrane for HPLC-MS analysis. Meanwhile, different

concentrations (0.001-5 mg L^{-1}) of the M8OI standards were prepared for M8OI quantitative analysis.

Reference

Habibul, N., Hu, Y.Y., Hu, Y., Sheng, G.P., 2021. Alkyl chain length affecting uptake of imidazolium based ionic liquids by ryegrass (Lolium perenne L.). Journal of Hazardous Materials 401. https://doi.org/10.1016/j.jhazmat.2020.123376

Rajeshkumar, S., Liu, Y., Zhang, X., Ravikumar, B., Bai, G., Li, X., 2018. Studies on seasonal pollution of heavy metals in water, sediment, fish and oyster from the Meiliang Bay of Taihu Lake in China. Chemosphere 191, 626–638. https://doi.org/10.1016/j.chemosphere.2017.10.078

Table S1. The change of Pb and M8OI content in treated water within one day.

Concentration (mg L^{-1})	Groups	Exposure time (h)				
		0	4	8	12	24
M8OI	Control	-	-	-	-	-
	Pb^{2+}	-	-	-	-	-
	M8OI	11.87 ± 0.51	11.62 ± 0.97	11.84 ± 2.40	11.44 ± 2.14	11.64 ± 0.85
	MIX	13.38 ± 1.08	11.11 ± 0.39	12.81 ± 0.17	12.67 ± 0.49	12.45 ± 0.55
Pb^{2+}	Control	-	-	-	-	-
	Pb^{2+}	9.82 ± 0.04	10.00 ± 0.5	10.86 ± 1.18	11.85 ± 0.55	10.22 ± 0.16
	M8OI	-	-	-	-	-
	MIX	9.82 ± 0.49	9.82 ± 0.41	10.26 ± 0.13	10.05 ± 0.35	9.53 ± 0.01

-, not detected.

Table S2. The details of primers sequences used for qPCR in this study.

Gene	Forward primer (5' to 3')	Reverse primer (5' to 3')	Length (bp)	Accession No.
GAPDH	TGGTTAAGGCTGCGGCTGAT	AGGTCACATACACGGTTGCTG T	199	XM_042740752.1
Claudin5	TGAAGGCGGAGAGCGTGAAG	GCGGGTTGTAGAAGTCGGAGA T	129	XM_042761641.1
Occludin	TTGACTCGGACCTCAGGACCTA	CTGCTACAGCCTGGTACTTGG A	129	XM_042729458.1
Zo-1	TGATGGTCAACGCCGTCTCC	TTCGTAGCTGTCGTCGTCCTC	179	XM_042752920.1

Table S3. The alteration of M8OI bioaccumulation in fish brain associated with exposure time.

	Exposure time （d）	7	14	28
M8OI (μg g^{-1} ww)	Control	-	-	-
	Pb^{2+}	-	-	-
	M8OI	91.19±24.50	172.47±34.08	199.15±16.27
	MIX	113.89±10.85	213.04±21.83	206.93±8.70

-, Not detected.

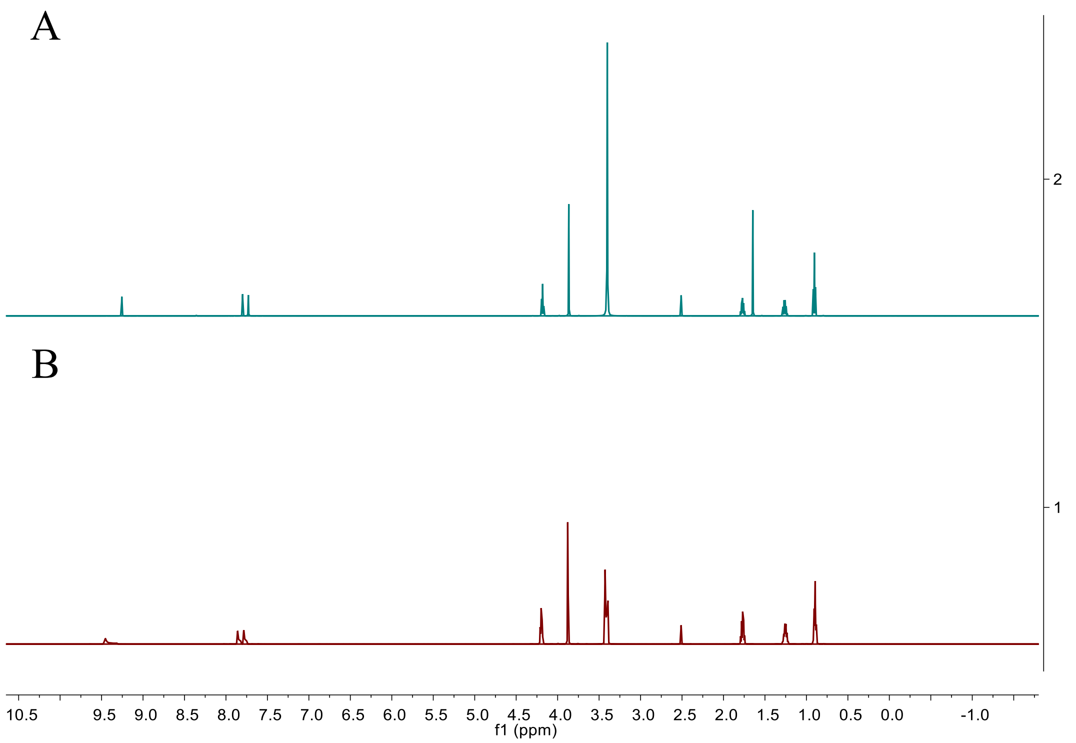

Figure S1. H-NMR spectrum of M8OI and Pb reaction in water at room temperature. (**A**) The H-NMR spectrum of Pb; (**B**) The H-NMR spectrum of Pb and M8OI reaction residue.

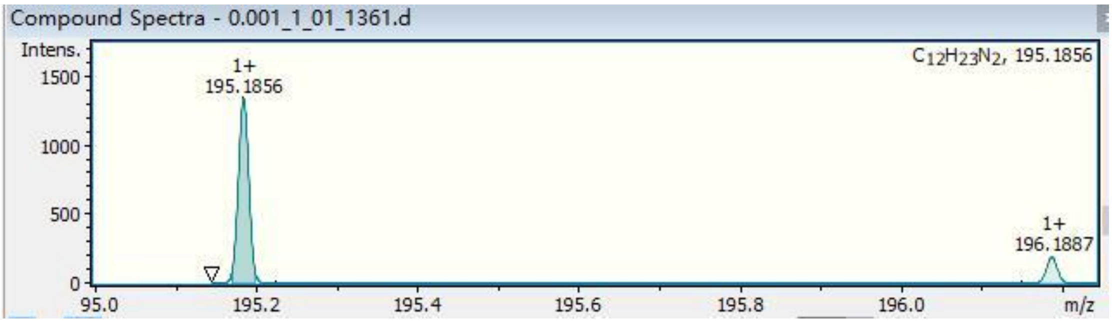

Figure S2. Selected LC-MS chromatographic data (m/z 195.19) of M8OI in standard solution and brain.

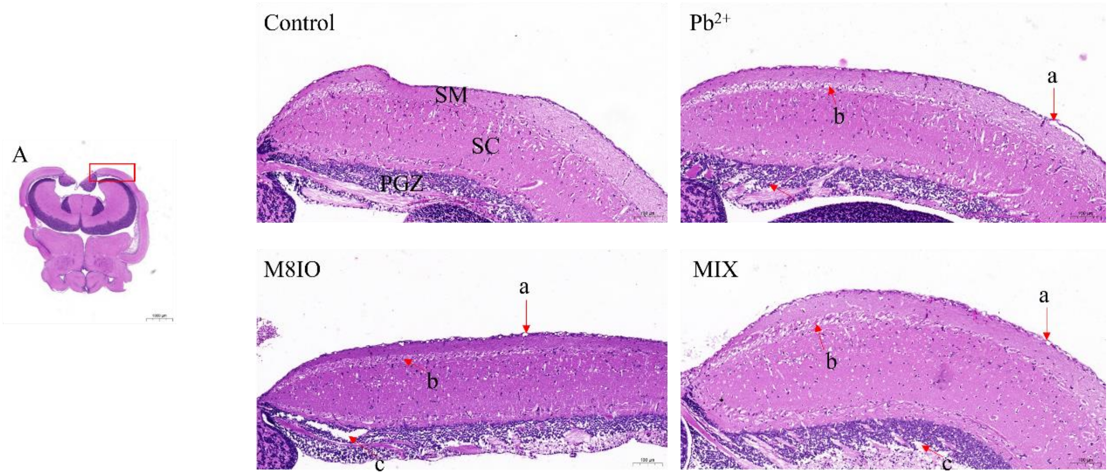

Figure S3. Histopathological analysis of common carp brain after exposure to Pb^{2+}, M8OI, and MIX for 28 d (scale bars= 100 μm). (A) Coronal sections in brain (scale bars=1000 μm). Note: SM, stratum marginale; SC, stratum centrale; PGZ, periglomerular gray zone; a, injured of pia mater. b, the gap between SM and SC. c, cavity of gray matter.
